# Maleimido-Functionalized NOTA Derivatives as Bifunctional Chelators for Site-Specific Radiolabeling

**DOI:** 10.3390/molecules16065228

**Published:** 2011-06-22

**Authors:** Christian Förster, Maik Schubert, Hans-Jürgen Pietzsch, Jörg Steinbach

**Affiliations:** Institute of Radiopharmacy, Helmholtz-Zentrum Dresden-Rossendorf, P.O. Box 510119, Dresden 01314, Germany

**Keywords:** 1,4,7-triazacyclonone-1,4,7-triacetic acid, NOTA, site-specific, maleimide, ethylene glycol spacer

## Abstract

Two basic and simple synthetic routes for mono- and bis-maleimide bearing 1,4,7-triazacyclononane-*N*,*N*’,*N*’’-triacetic acid (NOTA) chelators as new bifunctional chelators are described. The syntheses are characterized by their simplicity and short reaction times, as well as practical purification methods and acceptable to very good chemical yields. The usefulness of these two synthetic pathways is demonstrated by the preparation of a set of mono- and bis-maleimide functionalized NOTA derivatives. In conclusion, these two methods can easily be expanded to the syntheses of further tailored maleimide-NOTA chelators for diverse applications.

## 1. Introduction

1,4,7-Triazacyclononane-1,4,7-triacetic acid (NOTA) is an intensively investigated macrocyclic, multidentate chelator used for complexation of a broad variety of bi- and trivalent metal ions [1,2,3]. The size and geometry of the N_3_O_3_ coordination sphere seems to be perfect for Ga^3+^ ions due to the formation of a highly stable complex (log K ~ 31) [4]. The favorable complexation kinetics in aqueous solution, even at room temperature, spotlighted NOTA as chelating agent for gallium radionuclides for radiopharmaceutical experiments [5]. For covalent binding of NOTA to *in vivo* targeting entities, several modified NOTA chelators bearing reactive functional groups were developed. Such NOTA-based *bifunctional chelators* (BFC) possess either carboxyl, amino or isothiocyanate groups for conjugation reactions with various biologically active molecules [6,7,8].

However, a site-specific introduction of NOTA into proteins of high molecular weight (e.g., antibodies), which consist of large amounts of amino and carboxyl groups at their side chains, is very difficult or impossible. Such conjugation reactions afford conjugates with an average number of randomly located NOTA chelators per protein. Another disadvantage of this unpredictable NOTA distribution could be a decreased target affinity of the resulting protein-NOTA conjugates, particularly when NOTA is bound to target recognizing sites. Therefore, the introduction of NOTA via the thiol groups of cysteine residues could be more favorable. Accessible and reactive thiol groups provided by cysteine are often very rare compared to amino and carboxyl groups. For example, human serum albumin (HSA) is a protein consisting of 585 amino acids (M_w_ ~ 67 kDa) which can be modified via its amino, carboxyl, or thiol groups. The covalent binding of diethylene triamine pentaacetic acid (DTPA) chelators via HSA amino groups results in a broad statistical distribution with regard to binding position and the amount of bound DTPA. For instance, Wagner *et al.* were able to attach up to 17 DTPA units per HSA molecule [9]. In contrast, a modification via the intrinsic single thiol group resulted in well-defined conjugates using maleimide-functionalized reagents [10]. Another approach of site-specific labeling is based on the introduction of a cysteine into a cysteine-free proteinaceous targeting molecule. Such cysteine-free targeting vectors can easily be screened, identified and selected by phage-display technology. Tolmachev *et al.* are working with a recombinant anti-HER2 affibody for molecular imaging of human epidermal growth factor receptor type 2 (HER2) expressions in malignant tumors [11]. After introduction of a C-terminal cysteine residue into the cysteine-free anti-HER2 affibody scaffold (58 amino acids; ~7 kDa) the resulting conjugate was modified by a maleimide-bearing DOTA chelator and subsequently radiolabeled with ^111^In [12] as well as ^57^Co [13]. This site-specific labeling method afforded well-defined products and thus reliable and reproducible biodistribution data.

NOTA conjugates labeled with ^67^Ga, ^68^Ga, ^64^Cu, ^67^Cu, ^111^In, and potentially ^18^F via [18F]AlF [14] are suitable for diagnostic and therapeutical approaches. To our knowledge, only a few references referring to maleimide-functionalized NOTA chelating agents are known [15,16,17]. *Cox et al.* [15] described the syntheses of two mono-maleimido- and one bis-maleimido-functionalized NOTA derivatives. However, the lack of appropriate precursors necessitated a 10 steps synthetic route with an overall yield of less than 2% for the backbone modified NOTA-maleimide product. Eder *et al.* [16] presented a NOTA conjugated derivative of the vascular endothelial growth factor (VEGF). As spacing unit between the NOTA and VEGF moieties a large poly (ethylene glycol) was introduced. However, no information about the exact reaction conditions, yields, and analytical characterizations were given. A recently published work of Tolmachev *et al.* [17] emphasizes the necessity of maleimide derivatives of NOTA for site-specific labeling. Therefore, *N*-(2-aminoethyl)maleimide was coupled to one of the carboxyl groups of NOTA forming amide bond. The resulting MMA-NOTA product was bound to an anti-HER2 affibody having a single cysteine residue at the terminal C-position.

In the present work, we describe the convenient syntheses of a set of mono- and bis-maleimido-functionalized-NOTA chelators bearing different linkers and one or two maleimide entities in high chemical yields. The aim was to develop simple as well as effective synthetic routes to enable access to tailored maleimide-functionalized NOTA chelators with high flexibility in terms of molecular size, shape, and physicochemical properties of the resulting conjugates. The introduction of two maleimide groups has several advantages as well as further possibilities for applications: first, the binding probabilities are increased, which can be very helpful, especially when targeting molecules of high molecular weight. For smaller targeting molecules, bis-maleimide NOTA derivatives can be used to design radioactive, dimeric targeting structures to enhance their *in vivo* binding affinity due to the multivalency effect [18,19,20]. Second, such derivatives are suitable for grafting two different thiol-bearing species, e.g., a targeting molecule and a “pharmaceutical modifier”. Such modifiers are used for discrete manipulation and optimization of *in vivo* pharmacokinetics. In general, radioactive substances should be characterized by low blood circulation times to minimize radiation injury of healthy tissue. However, some radiolabeled compounds such as peptides or proteins of lower molecular size [21] show too rapid blood clearances. Schlesinger *et al.* [22,23] investigated radiolabeled, mirror-image L-oligonucleotides (L-ON) as a complementary system for tumor-pretargeting-approaches. Biodistribution studies showed that L-ON exhibit very low blood availabilities due to the polyanionic character and therefore fast renal excretion. To increase short *in vivo* half-lifes the usage of pharmaceutical modifiers are often applied [21,24,25,26]. Further important benefits of this strategy can be higher targeting rates as well as enhancement of the conjugate’s polarity to reduce liver uptake, lower immunogenicity, and minimization of unspecific tissue uptake.

## 2. Results and Discussion

**Scheme 1:**
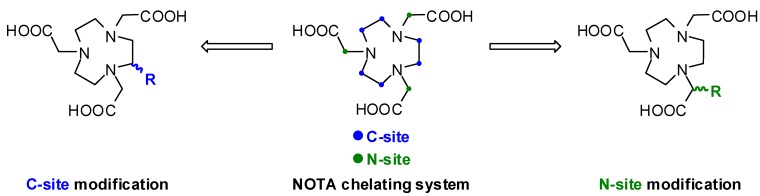
General possibilities of NOTA modification.

The maleimido-functionalized NOTA chelators were synthesized by using (*S*)-*p*-SCN-Bn-NOTA **1** and (*S*)-*p*-NH_2_-Bn-NOTA **2** as starting compounds due to their commercial availability and to keep the synthetic routes as short and simple as possible. Moreover, these two bifunctional NOTA chelators already have a modified backbone with functional groups (–NCS, –NH_2_) for the covalent binding of different maleimido-based residues. As pointed out in Scheme 1, there are two general possibilities for modifications of the NOTA to create bifunctional chelators: substitution of the backbone (C-site) or the methylene group of the acetic acid arm (N-site).

**Scheme 2:**
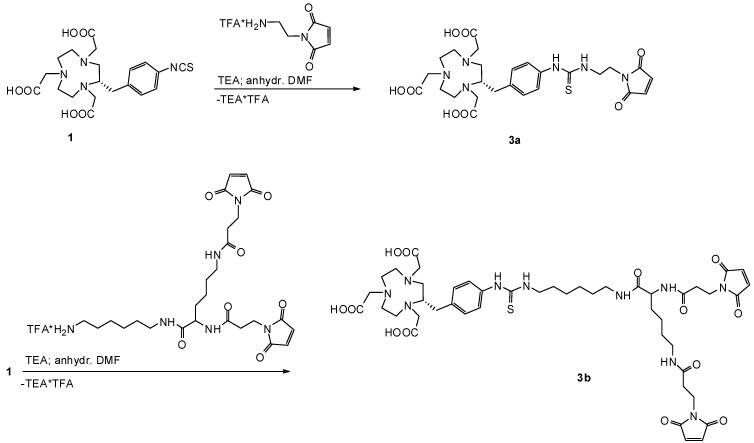
First reaction pathway for the syntheses of products **3a** and **3b**.

The first synthetic route we were investigating was based on derivatization of starting compound **1**. The isothiocyanate group was used for nucleophilic addition reactions with amino-functionalized mono- or bis-maleimide compounds to form stable thiourea bonds (Scheme 2).

**Scheme 3:**
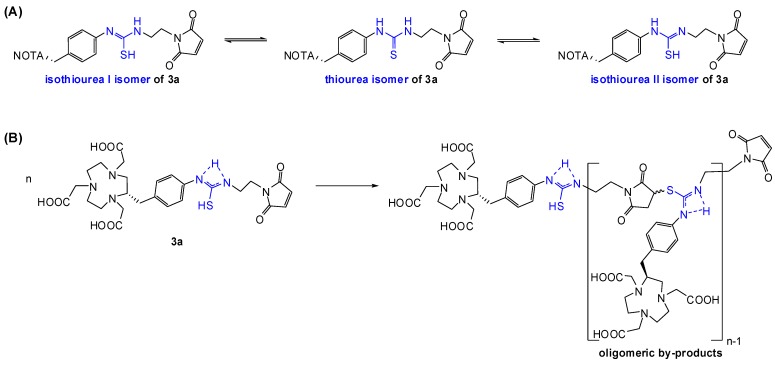
Tautomeric equilibrium (**A**) and oligomerization of product **3a** (**B**).

The presence of triethylamine (TEA) is necessary to transform the trifluoroacetic ammonium salts of both maleimide starting materials into reactive primary amino groups. To prevent side-reactions such as hydrolysis of maleimide or isothiocyanate groups, anhydrous DMF and TEA of the highest available quality was used. HPLC was utilized for isolation of the products to provide a very high purity which is required for this kind of application. A HPLC system based on a C-18 reverse-phase column and water as well as methanol (both containing 0.1 v/v % of TFA) as mobile phases gave optimal purification results. After isolation of and subsequent freeze-drying of **3a**, samples were taken and analyzed by analytical HPLC to assure its purity. Besides the expected product peak, additional peaks appeared in all chromatograms, so product samples were taken for mass spectrometric (ESI-MS and MALDI-TOF) analyses and the by-products were thus identified as oligomeric structures of **3a**. We also found that the amount of by-products was proportional to the time between HPLC isolation and freeze-drying. This indicates that the formation of by-products was related to the HPLC eluents. As mentioned above, 0.1 v/v % of TFA in both mobile phases was used to prevent maleimide hydrolysis during and after HPLC purification. Moreover, acidic conditions provided best purification and isolation results in terms of sufficient differences in retention times. Unfortunately, TFA apparently shifts the tautomeric equilibrium of the thiourea bond towards the two isothiourea isomers **I** and **II** (Scheme 3A). The reactive isothiourea-thiol-groups undergo nucleophilic addition reactions with maleimide groups resulting in the observed oligomeric products (Scheme 3B).

To minimize the mentioned side-reactions, the product fractions were shock-frozen with liquid nitrogen immediately after collecting from HPLC and subsequently freeze-dried. Unfortunately, this approach was characterized by a lower reproducibility concerning the amount of by-products, so we decided to use 0.1 v/v % of acetic acid instead of TFA in both mobile phases. These HPLC conditions in combination with shock-freezing immediately after HPLC isolation the side-reactions were prevented. Following this procedure, we were able to synthesize and characterize the mono-maleimido-NOTA chelator **3a** as well as the bis-maleimido-NOTA chelator **3b**.

**Scheme 4:**
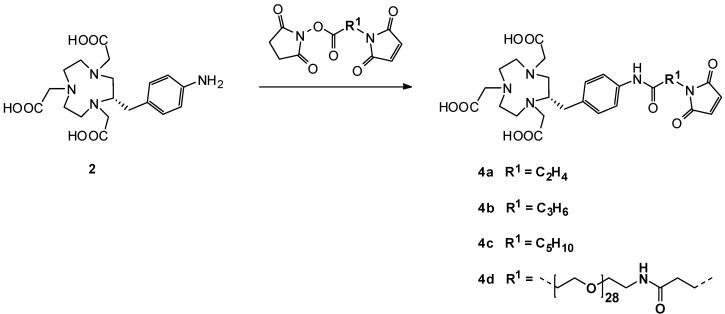
Second reaction pathway for the syntheses of **4a–4d**.

Only the two amino-group-bearing maleimide compounds presented above were commercially available. To overcome this pitfall and to prevent additional syntheses of appropriate maleimide starting compounds, we started to work out another strategy by modifying NOTA compound **2** for the syntheses of NOTA-maleimides **4a–4d** (Scheme 4).

Several maleimide derivatives with *N*-hydroxysuccinimidyl esters and different spacers are available. We have chosen three aliphatic, heterobifunctional cross-linkers as well as an oligo(ethylene glycol) (OEG) derivative containing 28 ethylene glycol units. Different spacer lengths between targeting molecule and labeling site are often necessary to preserve the high target affinity of the native molecule [19,20]. Moreover, we found that variation of linker length influences radiolabeling efficiency (unpublished results). We also synthesized NOTA-OEG_28_-Mal **4d**, because we purposefully wanted to influence the biodistribution properties of our targeting vectors (L-ON for pretargeting-approaches) by using this large spacer. Modifications of biological active molecules with OEG or poly(ethylene glycol) (PEGylation) is a commonly applied procedure to alter pharmacokinetic parameters such as the solubility of (radio-)pharmaceuticals [25,26]. This strategy of a simultaneous modification of targeting molecules within one chemical reaction can be very useful to save time and costs. Relating to our scientific interests, the OEG size of 28 units seemed to be suitable, but this concept can easily be adjusted to bigger as well as smaller OEG spacers according to the size and shape of the targeting molecule. Several Mal-OEG_x_/PEG-NHS esters are commercially available to gain tailored NOTA-OEG/PEG-Mal derivatives (e.g., x = 2, 4, 6, 8, 24, 27 as well as up to several kDa sizes).

The product identification and isolation by HPLC was challenging because of nearly equal retention times of maleimide-OEG_28_-NHS ester, product **4d**, and the by-products formed by hydrolysis reactions of Mal-OEG_28_-NHS ester and **4d**. Using either TFA or acetic acid conditions no satisfying separations were possible. Therefore, different buffer solutions with various pH values were tested as mobile phases. Good results were obtained with triethylammonium acetate at pH = 5.0. The HPLC product peak was identified by taking reaction samples to which three different chemicals were added. The product peak shifted after the addition of GaCl_3_, sodium hydroxide or HS-OEG_8_-OMe (purchased from IRIS Biotech). The complexation of Ga^3+^ ions served as indicator for the presence of NOTA, sodium hydroxide (inducing ring opening by formation of corresponding maleic acid derivative) and HS-OEG_8_-OMe (nucleophilic addition) as indicators for the presence of the maleimido group.

Short reaction times and high chemical yields were obtained when performing the reactions in DMF in addition to phosphate buffered solution (1:1 v/v). DMF was necessary to dissolve the poorly water-soluble maleimide starting compounds (except for the synthesis of **4d**). The weak acidic conditions (phosphate buffer, pH = 6.6) very effectively minimized maleimide hydrolysis of the formed products during the reaction procedures. On the other hand, at this pH value the aromatic amino group of **2** is not protonated to allow reactions with NHS ester groups of the maleimide derivatives. An excess of maleimide reagents relative to **2** was necessary, because even at weakly acetic conditions NHS esters are rapidly hydrolyzed into their corresponding non-reactive carboxyl groups. Unfortunately, we could not develop an appropriate HPLC method to isolate product **4c** without contamination by the starting maleimide compound. The maleimide-NHS ester was always exhibiting nearly the same retention time as the product **4c**. Therefore, investigations using different HPLC columns for better separations have to be undertaken. In contrast to all synthesized products, the use of ^1^H-NMR to characterize compound **4d** was not conclusive because signals were strongly overlapped in the NMR spectrum.

Initial reactions using anhydrous DMF as solvent without the addition of phosphate buffer and TEA as base to convert the aromatic ammonium chloride salt of **2** into a more reactive amino group, gave unsatisfying chemical yields and very long reaction times (up to two weeks). The poor chemical yields could be ascribed to formation of hydroxyl anions caused by the presence of water in the NOTA starting material **2** (≤10 mass% of H_2_O according to supplier; **2** was delivered as *p*-NH_2_-Bn-NOTA*4HCl), which undergo hydrolysis reactions of NHS ester and maleimide residues.

**Scheme 5:**
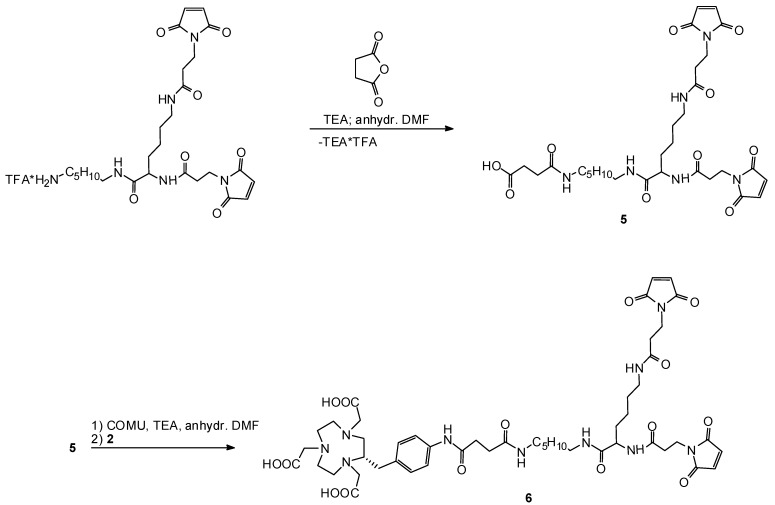
Synthesis of NOTA-bis-mal product **6**.

We also synthesized a bis-maleimide derivative **6** (Scheme 5). Therefore, the bis-maleimide starting compound was reacting with succinic anhydride to introduce a carboxyl group (compound **5**). In the next step, a reactive ester of **5** was generated *in-situ* and subsequently coupled to starting compound **2**. Unsuccessfully, we tried to synthesize and to isolate the NHS ester of **5** in different ways. Alternatively, we were investigating several in situ-coupling reagents such as *N*-(3-dimethylamino-propyl)-*N*-ethylcarbodiimide hydrochloride (EDC**·**HCl), *N*,*N*,*N*′,*N*′-tetramethyluronium-*O*-(*N*-succinimidyl)tetrafluoroborate (TSTU), *N,N,N’,N’-*tetramethyl-*O-*(1*H*-benzotriazol-1-yl)uroniumhexa- fluorophosphate (HBTU), *N,N,N′,N′*-tetramethyl-*O*-(7-azabenzotriazol-1-yl)uronium hexafluoro-phosphate (HATU). Best results were found when using (1-cyano-2-ethoxy-2-oxoethylidene-aminooxy)-dimethylaminomorpholinocarbenium hexafluorophosphate (COMU). Products **4a–4d**, **5** and **6** were also purified and isolated by HPLC techniques. As expected, no acid-induced side-reactions after HPLC separation of these products occurred.

## 3. Experimental

### 3.1. Materials

Chemical reagents and solvents were purchased from commercial sources in the highest available purity and were used without further purification. (*S*)-*p*-SCN-Bn-NOTA (**1**) as well as (*S*)-*p*-NH_2_-Bn-NOTA (**2**) were purchased from Macrocyclics (Dallas, TX, USA). The maleimide starting materials were purchased from IRIS Biotech (Germany). Reactions were performed in 0.5, 1.5, and 2.0 mL DNA low-binding tubes (Eppendorf, Germany) using a Thermomixer (Eppendorf, Germany). ^1^H-NMR (400 MHz) were recorded at room temperature on a Varian Inova 400 spectrometer using the residual proton resonance in deuterated water (D_2_O) as reference. MALDI-TOF spectra were obtained with a Micromass Tandem Quadropole instrument. The MALDI-TOF matrix, 3-hydroxypicolinic acid (3-HPA), was purchased from Bruker. The matrix was a mixture of 3-HPA (50 mg/mL in ultrapure water and CH_3_CN 1:1) and diammonium hydrogen citrate (100 mg/mL) with a ratio of 10:1. For ESI-MS-spectrometry all products were dissolved in Ultra-pure water.

### 3.2. Chromatographic Purifications and Analyses

HPLC analyses were performed with following equipment and methods: The HPLC columns used were: (A) Eurospher 100 C_18_ column (Knauer, Germany), 5 μm, 4 × 250 mm; (B) Eurospher 100 C_18_ column (Knauer, Germany), 5 μm, 8 × 250 mm. Method A1: flow 1 mL/min, UV 220 nm, water-methanol (90/10 v/v %) containing 0.1 v/v % acetic acid (a) and water-methanol (10/90 v/v %) containing 0.1 v/v % acetic acid (b), gradient (a): 0 min 100 %, 0–20 min 0%. Method A2: flow 1 mL/min, UV 220 nm, water-methanol (90/10 v/v %) containing 0.1 vol % trifluoroacetic acid (TFA) (a) and water-methanol (10/90 v/v %) containing 0.1 v/v % TFA (b), gradient (a): 0 min 100%, 0–20 min 0%. Method A3: flow 1 mL/min, UV 220 nm, water containing 0.1 v/v % TFA (a) and water-acetonitrile (30/70 v/v %) containing 0.1 v/v % TFA (b), gradient (a): 0 min 100%, 0–36 min 49%. Method A4: flow 1 mL/min, UV 220 nm, water-acetonitrile (90/10 v/v %) containing 1 mM triethyl-ammonium acetate (TEAA; pH = 5.0) (a) and water-acetonitrile (10/90 v/v %) (b), gradient (a): 0 min 100%, 0–25 min 50%. Method B1: flow 3 mL/min, UV 254 nm, water-methanol (90/10 v/v %) containing 0.1 vol % acetic acid (a) and water-methanol (10/90 v/v %) containing 0.1 v/v % acetic acid (b), gradient (a): 0 min 100%, 0–30 min 0%. Method B2: flow 3 mL/min, UV 220 nm, water-methanol (90/10 v/v %) containing 0.1 vol % TFA (a) and water-methanol (10/90 v/v %) containing 0.1 v/v % TFA (b), gradient (a): 0 min 100 %, 0–30 min 0%. Method B3: flow 3 mL/min, UV 220 nm, water containing 0.1 vol % TFA (a) and water-acetonitrile (30/70 v/v %) containing 0.1 vol % TFA (b), gradient (a): 0 min 100%, 0–56 min 49%. Method B4: flow 3 mL/min, UV 220 nm, water-acetonitrile (90/10 v/v %) containing 1 mM triethyl-ammonium acetate (TEAA; pH = 5.0) (a) and water-acetonitrile (10/90 v/v %) (b), gradient (a): 0–5 min 100%, 5–40 min 50%.

### 3.3. General Procedure for the Syntheses of Thiourea Bound NOTA-Maleimides

To a solution of **1** (7.8 mg, 15 μmol) in anhydrous dimethylformamide (DMF, 100 μL) triethylamine (8.5 μL, 60.8 μmol) was added. The corresponding ammonium trifluoroacetate maleimide (15.8 μmol) was dissolved in anhydrous DMF (100 μL) and added dropwise to the solution of **1**. The reaction mixture was incubated at 25 °C for 2 h. Acetic acid (21 μL, 369 μmol) was added and the reaction mixture was fractionized by HPLC. HPLC-fractions containing the desired product were immediately shock-frozen with liquid nitrogen and lyophilized.

*(S)-2-{2-(4-{3-[2-(2,5-dioxo-2,5-dihydro-(1H)-pyrrole-1-yl)ethyl]thiourea}benzyl)-4,7-bis(carboxy-methyl)-1,4,7-triazacylononane}acetic acid* (**3a**). HPLC method A1: t_R_ = 11.4 min. HPLC method B1: t_R_ = 14.5 min. Yield: 3.3 mg (38%; white powder). ESI-MS *m/z* 591 [M+H]^+^, 613 [M+Na]^+^. ^1^H-NMR: 2.92–4.03 (m, 23H), 7.00 (s, 2H, CH=CH), 7.32 (d, *J* = 7.6 Hz, 2H, aromatic CH), 7.51 (d, *J* = 7.6 Hz, 2H, aromatic CH).

*(S)-2-(2-{4-[3-(6-{2,6-bis[3-(2,5-dioxo-2,5-dihydro-1H-pyrrole-1-yl)propanamido]-hexanamido}-hexyl)thiourea]benzyl}-4,7-bis(carboxymethyl)-1,4,7-triazacylononane)acetic acid* (**3b**). HPLC method A1: t_R_ = 17.3 min. HPLC method B1: t_R_ = 19.9 min. Yield: 4.5 mg (28%; white powder). ESI-MS *m/z* 510 [M+H+Na]^2+^, 521 [M+2Na]^2+^, 997 [M+H]^+^, 1019 [M+Na]^+^, 1035 [M+K]^+^. ^1^H-NMR: 1.09–1.60 (m, 14H, CH_2_), 1.91 (s, 3H, CH_3_COOH), 2.28–2.41 (m, 4H, CH_2_CONH), 2.60–3.73 (m, 29H), 3.93 (t, *J* = 7.2 Hz, 1H, CH), 6.68 (s, 4 H, CH=CH), 7.06 (d, *J* = 7.6 Hz, 2H, aromatic CH), 7.18 (m, 2H, aromatic CH).

### 3.4. General Procedure for the Syntheses of Amide Bound NOTA-Maleimides

To a solution of **2** (2.8 mg, 5 μmol) in phosphate buffer (150 μL, pH = 6.6) the corresponding *N*-hydroxysuccinimide ester functionalized maleimide (30 μmol, dissolved in 150 μL DMF) was added. The slightly yellow dispersion was incubated at 25 °C for 1.5 h. The reaction mixture was centrifuged and the supernatant was taken for HPLC purification. HPLC-fractions containing the product were lyophilized.

*(S)-2-(2-){4-[3-(2,5-dioxo-2,5-dihydro-(1H)-pyrrole-1-yl)propanamido]benzyl}-4,7-bis(carboxy-methyl)-1,4,7-triazacylononane)acetic acid* (**4a**). HPLC method A2: t_R_ = 12.7 min. HPLC method B2: t_R_ = 15.3 min.Yield: 2.4 mg (86%; white powder). ESI-MS *m/z* 560 [M+H]^+^, 582 [M+Na]^+^. ^1^H-NMR: 2.53 (t, *J* = 6.2 Hz, 2H, CH_2_CONH), 2.60–3.70 (m, 19H), 3.74 (t, *J* = 6.2 Hz, 2H, CH_2_N), 6.70 (s, 2H, CH=CH), 7.10–7.30 (m, 4H, aromatic CH).

*(S)-2-(2-{4-[4-(2,5-dioxo-2,5-dihydro-(1H)-pyrrole-1-yl)butanamido]benzyl}-4,7-bis-(carboxymethyl)-1,4,7-triazacylononane)acetic acid* (**4b**). HPLC method A2: t_R_ = 13.6 min. HPLC method B2: t_R_ = 14.8 min. Yield: 2.4 mg (83%; white powder). ESI-MS *m/z* 574 [M+H]^+^, 596 [M+Na]^+^, 612 [M+K]^+^. ^1^H-NMR: 1.86 (q, *J* = 6.6 Hz, 2H, CH_2_CH_2_CH_2_), 2.29 (t, *J* = 6.6 Hz, 2H, CH_2_CO), 2.40–3.80 (m, 21H), 6.62 (s, 2H, CH=CH), 7.10–7.26 (m, 4H, aromatic CH).

*(S)-2-(2-{4-[6-(2,5-dioxo-2,5-dihydro-(1H)-pyrrole-1-yl)hexanamido]benzyl}-4,7-bis-(carboxymethyl)-1,4,7-triazacylononane)acetic acid* (**4c**). HPLC method A2: t_R_ = 14.8 min. HPLC method B2: t_R_ = 16.3 min. Yield: 2.6 mg (85%; white powder). ESI-MS *m/z* 602 [M+H]^+^, 624 [M+Na]^+^ (the product contains small amounts of 6-(maleimido)hexanoic acid N-hydroxysuccinimide ester due to insufficient separation capacity).

*(S)-2-[2-(4-{87-[2-(2,5-dioxo-2,5-dihydro-(1H)-pyrrole-1-yl)ethanamido]-4,7,10,13,16,19,22,25,28,31,34,37,40,43,46,49,52,55,58,61,64,67,70,73,76,79,81,84-octacosaoxa-heptaoctacontan}benzyl)-4,7-bis-(carboxymethyl)-1,4,7-triazacylononane]acetic acid* (**4d**): To a solution of **2** (2.0 mg, 3.6 μmol) in 200 μL phosphate buffer (pH = 6.6) Mal-OEG_28_-NHS ester (10.0 mg, 6.4 μmol) was added. The slightly yellow solution was incubated at 26 °C for 2.0 h. Product 4d was isolated by HPLC separation. HPLC-fractions containing product **4d** were lyophilized to obtain **4d** as a yellow-white powder. HPLC method A4: t_R_ = 20.4 min. HPLC method B4: t_R_ = 32.4 min. Yield: 4.7 mg (50%; white powder). ESI-MS *m/z* 944 [M+H+Na]^2+^, 955 [M+2Na]^2+^. MALDI-TOF *m/z* 933 [M+2H]^2+^, 1864 [M+H]^+^, 1886 [M+Na]^+^, 1902 [M+K]^+^.

*4-(6-{2,6-bis[3-(2,5-dioxo-2,5-dihydro-(1H)-pyrrole-1-yl)propanamido]hexanamido}hexylamino)-4-oxobutanoic acid* (**5**). To a solution of the bis-maleimide starting compound Bis-mal-Oc-NH_2_*TFA (20 μmol, 13.2 mg) in anhydrous DMF-TEA (100 μL, 20 μmol, 2.0 mg, 2.8 μL) was added. Succinic anhydride (23 μmol, 2.3 mg) was dissolved in anhydrous DMF (70 μL) and added dropwise. The reaction mixture was incubated at 26 °C for 30 min. After the addition of TFA (40 μmol, 4.6 mg, 3 μL) to the colorless solution, the reaction mixture was fractionized by HPLC. HPLC-fractions containing product **5** were lyophilized to obtain **5** as a white powder. Yield: 8.1 mg (63%; white powder). ESI MS (*m/z*): 647 [M+H]^+^, 669 [M+Na]^+^, 685 [M+K]^+^, 645 [M−H]^−^. HPLC method A2: t_R_ = 13.0 min. HPLC method B2: t_R_ = 17.5 min. ^1^H-NMR: 1.32–1.50 (m, 6H, CH_2_), 1.52–1.66 (m, 6H, CH_2_CH_2_NH), 1.72–1.85 (m, 2H, CH_2_CH), 2.59–2.78 (m, 8H, CH_2_CO), 3.20–3.35 (m, 6H, CH_2_NH), 3.88–3.95 (m, 4H, CH_2_N), 4.21 (t, *J* = 7.0 Hz, 1H, CH), 6.99 (s, 4H, CH=CH), 8.13 (t, *J* = 4.0 Hz, 3H, NH) 8.40 (d, *J* = 7.2 Hz, 1H, NH).

*(S)-2-(2-{4-[4-(6-{2,6-bis[3-(2,5-dioxo-2,5-dihydro-1H-pyrrol-1-yl)-propanamido]-hexanamido}-hexylamino)-4-oxobutanamido]benzyl}-4,7-bis-(carboxymethyl)-1,4,7-triazacylononane)acetic acid* (**6**). Compound **5** (6.7 μmol) was dissolved in anhydrous DMF and TEA (120 μL, 6.7 μmol, 0.7 mg, 0.9 μL) was added. An anhydrous DMF solution (35 μL) of COMU (5.4 μmol, 2.3 mg) was added dropwise. After reaction time for a maximum of 40 min at 26 °C, a solution of **2** (1.9 mg, 3.4 μmol), dissolved in DMF (55 μL) and TEA (2 μL, 13.5 μmol, 1.4 mg), was added. The reaction mixture was incubated at 26 °C over night. After addition of TFA (40.4 μmol, 4.6 mg, 3 μL) the reaction mixture was fractionized by HPLC. HPLC-fractions containing product **6** were lyophilized to obtain **6** as a white powder. Yield: 1.46 mg (42 %; white powder). ESI MS (*m/z*): 520 [M + 2H]^2+^, 531 [M + H + Na]^2+^, 1038 [M + H]^+^, 1060 [M + Na]^+^. HPLC method A3: t_R_ = 31.8 min. HPLC method B3: t_R_ = 51.8 min. ^1^H-NMR: 1.20–1.34 (m, 6H, CH_2_), 1.37–1.48 (m, 6H, CH_2_CH_2_NH), 1.58–1.74 (m, 2H, CH_2_CHNH), 2.45–2.78 (m, 8H, CH_2_CONH), 2.80–4.02 (m, 2H), 4.1 (t, *J* = 7.0 Hz, 1H, CH), 6.85 (s, 4H, CH=CH), 7.30 (d, *J* = 8.0 Hz, 2H, aromatic CH), 7.40 (d, *J* = 8.4 Hz, 2H, aromatic CH).

## 4. Conclusions

We have developed two basic and simple synthetic pathways for the preparation of a variety of new mono- and bis-maleimido-NOTA chelators. The first route is based on nucleophilic addition reactions of *p*-SCN-Bn-NOTA **1** and maleimide starting materials with primary amino groups under formation of thiourea bonds. In the second pathway, maleimide derivatives with reactive carboxylic acid ester residues and *p*-NH_2_-Bn-NOTA **2** were coupled to form amide bound products. The practicability of these two methods is demonstrated by the preparation of a set of different derivatives **3a–3b**, **4a–4d** and **6**. The isolation of thiourea bond-bearing products **3a** and **3b** was initially difficult due to side-reactions after HPLC separation. The side-reactions were suppressed by increasing the pH value of both mobile phases during HPLC purification.

We also synthesized a derivative **4d**, which is characterized by a very long spacer consisting of 28 ethylene glycol units. Such compounds can be used to enable site-specific radiolabeling and optimization in terms of blood circulation half life, enhanced targeting rates, conjugate polarity, lower immunogenicity, or minimization of unspecific tissue uptake. Several maleimide compounds with reactive NHS ester and different OEG- or PEG-spacer lengths are commercially available to allow the preparation of suitable derivatives according to individual requirements.

The described synthetic methods can be transferred to any other amino group or active ester bearing maleimide starting material. This enables access to tailored bifunctional NOTA-maleimide chelators for modification and subsequent radiolabeling of potential targeting molecules.

Prospective investigations include the conjugation of selected NOTA-maleimide products with target-specific compounds as well as the evaluation of their radiolabeling ability and *in vivo* behavior. Similarly, other chelating systems will be modified by utilization of the developed synthetic methods.
